# First field evaluation of the optimized CE marked Abbott protocol for HIV RNA testing on dried blood spot in a routine clinical setting in Vietnam

**DOI:** 10.1371/journal.pone.0191920

**Published:** 2018-02-09

**Authors:** Fabien Taieb, Tram Tran Hong, Hien Thi Ho, Binh Nguyen Thanh, Tram Pham Phuong, Dung Viet Ta, Nhung Le Thi Hong, Hien Ba Pham, Lan Thi Huong Nguyen, Huong Thi Nguyen, Thu Trang Nguyen, Edouard Tuaillon, Eric Delaporte, Huong Le Thi, Hau Tran Thi Bich, Tuan Anh Nguyen, Yoann Madec

**Affiliations:** 1 Emerging Diseases Epidemiology Unit, Institut Pasteur, Paris, France; 2 Center of Translational Research, Institut Pasteur, Paris, France; 3 National Institute of Hygiene and Epidemiology, Virology laboratory, HIV/AIDS Department, Hanoi, Vietnam; 4 Hanoi University of Public Health, HIV/AIDS Prevention and Control department, Hanoi, Vietnam; 5 Dong Da General Hospital, outpatient clinic, Hanoi, Vietnam; 6 Dong Anh outpatient clinic, Hanoi, Vietnam; 7 Nam Tu Liem outpatient clinic, Hanoi, Vietnam; 8 INSERM U1058 Unit: Pathogenesis and Control of Chronic Infections, Montpellier University Hospital, Montpellier, France; 9 UMI 233 TransVIHMI, IRD and Université de Montpellier 1, Montpellier, France; 10 Vietnam Administration of HIV/AIDS Control, Hanoi, Vietnam; 11 Provincial AIDS Center of Hanoi, Hanoi, Vietnam; Public Health Agency of Canada, CANADA

## Abstract

**Background:**

Viral load (VL) monitoring of HIV-infected patients in decentralized areas is limited due to logistic constraints. Dried Blood Spots (DBS) offer the opportunity to collect samples in remote area which can be easily transferred and tested at a central laboratory. The MOVIDA (Monitoring Of Viral load In Decentralized Area) project evaluated the performance of VL measurements on DBS using the new CE marked optimized Abbott protocol.

**Methods:**

HIV-1 infected adults from three outpatient clinics in Hanoi (Vietnam) were enrolled into the study between 1 March and 13 April 2017. VL was measured on DBS using the optimized protocol provided by the manufacturer and compared to plasma VL as reference method on the Abbott m2000rt RealTime HIV-1 platform. Sensitivity was defined as the ability for DBS samples to correctly identify VL failure at the threshold of 1000 copies/mL of plasma, while specificity represented the ability to identify patients with a plasma HIV-RNA VL of <1000 copies/mL.

**Results:**

A total of 203 patients were enrolled in the study, of which 152 (75%) were male. Median age was 38 [inter quartile range: 34–43] years. Of these patients, 37 were untreated, 38 on ART for <6 months and 117 were on ART for ≥6 months. A strong correlation between VL results in plasma and from DBS was observed (ρ = 0.95; p<0.001). Plasma VL was ≥1000 copies/mL in 71 patients. The sensitivity of DBS was 90.1% (95% confidence interval [CI]: 80.7–95.9) and the specificity was 96.2% (95% CI: 91.4–98.8).

**Conclusions:**

The new optimized Abbott DBS protocol performed well in this study, meeting the WHO performance criteria for the use of DBS for HIV VL monitoring. Scaling up VL monitoring using DBS can be used to reach the last 90 in the UNAIDS targets of 90-90-90 to help end the AIDS epidemics. However, sensitivity remains the main challenge for manufacturers to prevent maintaining patients in virological failure on inefficient ART.

## Introduction

In 2014, the UNAIDS launched their 90-90-90 treatment targets to end the AIDS epidemics [1].This means that by 2020, 90% of all people living with HIV should know their HIV status, 90% of all people with diagnosed HIV infection should receive sustained antiretroviral therapy (ART), and 90% of all people receiving antiretroviral therapy should have viral suppression.

Virological monitoring is crucial for patients on ART to accurately detect virological failure, and to modify ART in a timely manner. Clinical and immunological criteria have been shown to poorly predict virological failure [2–4]. The World Health Organization (WHO) currently recommends viral load (VL) monitoring at 6 months and 12 months after ART initiation and thereafter annually [5]. While VL monitoring is the standard of care in developed countries, its availability remains limited in many settings, often constrained by cost, technologies, human resources, etc. [6]. In remote areas, distance from sufficiently equipped laboratories able to perform VL measurements and sometimes availability of electricity further limit the use of VL.

To overcome the difficulties of VL monitoring in remote areas, several strategies have recently emerged to complement the existing approaches. Point-of-care (POC) devices have been developed, which allow rapid, on-site VL measurements [7]. POC devices show encouraging results [8, 9], but still require laboratory capacities. Dried blood spots (DBS) using venous or capillary whole blood has been included in the latest WHO recommendations [5]. However, the ability to diagnose virological failure using DBS still needs to be validated before being scaled up. Several studies conducting such evaluation have been published [10–13], with variable results.

Abbott Molecular has developed a semi-automated two-spot HIV-1 DBS protocol which has been used in a number of clinical research studies [10, 11, 13–16]. A comprehensive review of studies evaluating and comparing the performance of DBS samples to plasma samples for HIV VL quantification demonstrated that DBS assays can achieve sensitivity and specificity above 90%. To increase ease-of-use and to improve the assay performance, a one-spot DBS protocol for the Abbott RealTime HIV-1 assay was developed, this protocol was CE marked in 2016 [17].

The current study aimed to evaluate the sensitivity and specificity of the CE marked optimized DBS protocol in identifying patients in virological failure, defined by a VL above the threshold of 1000 copies/mL, in routine clinical settings using a laboratory in Hanoi, Vietnam, as compared to VL in plasma samples.

## Methods

HIV-1 infected adults from three outpatient clinics (OPCs) in Hanoi (Vietnam) were invited to participate in the study. We anticipated that most patients on ART for≥6 months would exhibit low VL [18–20], patients on ART for <6 months would essentially present intermediate VL, while pre-ART patients would present high VL. Thus, in order to obtain a wide range of VL levels, pre-ART patients, patients on ART for <6 months and patients on ART for ≥6 months were included in the study.

Patients were given detailed information (oral and written) about the study, after which they decided to take part or not in the study. Patients who provided written informed consent were enrolled and were randomly assigned an identification number. A case-report form was completed by the clinician recording the gender and age of the patient and ART history. A whole blood sample was drawn from venipuncture, collected in EDTA tube and transferred in a cool box the same day (within 3 hours following sampling) to the HIV/AIDS molecular laboratory of the National Institute of Hygiene and Epidemiology (NIHE) in Hanoi (Vietnam) where plasma and DBS samples were processed, and VL measurements were performed.

At the HIV/AIDS molecular laboratory, 70μl of fresh whole blood were dropped on each of the 5 one-half-inch (12 millimeter) circles of two perforated Munktell TFN paper cards (DBS) using a calibrated pipette. DBS were then left to dry at ambient temperature for 3 hours before being packed individually in ziplock bags with two desiccants. DBS cards were maintained at ambient temperature for 2 weeks, after which they were frozen at -20°C following the WHO guidelines. This was done in order to mimic real life settings where delays between DBS collection, reception at a central laboratory and VL measurement often occur. The remaining whole blood was centrifuged at 2000–2500 rpm for 10 minutes and two plasma samples of 2mL were collected in cryotubes and frozen at -80°C until VL was measured.

To ensure VL measurements from plasma and from DBS for the same patient were blinded, pre-identified stickers with randomly allocated identification numbers for EDTA tubes, DBS cards and plasma cryotubes were used. The correspondence list was created and stored by investigators at Institut Pasteur (Paris, France).

### VL measurement on plasma

VL was quantified using the Abbott real-time HIV-1 PCR with 0.8 mL of plasma in the m2000rt system following the manufacturer’s recommendations. After automated nucleic acid extraction, a volume of 50 μL was used for PCR. The lower detection limit was 40 copies/mL.

### VL measurement on DBS

NIHE used the Open Mode HIV-1 RNA Quantitative DBS protocol file for HIV VL testing. Following the manufacturer’s recommendations, one single DBS spot of 70μL per patient was placed into a tube with 1.3 mL of mDBS buffer (List No. 09N02-001). The tubes were mixed by swirling and incubated for 30 minutes in a heating block (Benchmark Scientific) at 55°C. Samples were directly loaded on the m2000sp platform without additional pre-analytical manipulation. Open mode 1.0 mL HIV-1 RNA DBS IUO TT version 11 protocol was used for extraction and amplification. The Abbott HIV-1 RealTime assay uses automated extraction of viral nucleic acid on the m2000sp using purification reagents and purification process specific for RNA. The lower detection limit of the assay indicated by the manufacturer was 839 copies/mL.

### Statistical analysis

Plasma VL results were considered as the gold standard. Plasma VL measurements below the threshold were arbitrarily attributed the value 20 copies/mL. DBS VL results below the detection limit were also attributed the value 20 copies/mL.

Correlation between log-transformed plasma and DBS VL measurements was estimated. A bland-Altman analysis was conducted to evaluate the concordance between the two methods.

Sensitivity and specificity with 95% confidence intervals (CI) of DBS to identify virological failure at the threshold of 1000 copies/mL were measured. Sensitivity was estimated as the proportion of plasma VL ≥1000 copies/mL for which DBS VL was also ≥1000 copies/mL. Specificity was estimated as the proportion of plasma VL <1000 copies/mL for which DBS VL was also <1000 copies/mL. The Kappa coefficient was used to measure agreement between plasma and DBS measurements.

### Ethical consideration

The study protocol was approved by the Institutional Review Board from the Hanoi School of Public Health (Hanoi, Vietnam) (decision number 333/2016/YTCC-HD3), and the Institutional Review Board from Institut Pasteur (decision number 2016–05 IRB/1). Authorization for data processing has been obtained from French legal authority (Commission Nationale Informatique et Liberté, decision number DR-2017-046). Only participants providing written informed consent were enrolled, and random anonymous identification numbers were assigned to each patient to guarantee confidentiality.

## Results

A total of 203 patients were enrolled in the study between March 1^st^ and April 13^th^ 2017 in three OPCs in Hanoi. Of these patients, 152 (74.9%) were male and the median (inter quartile range (IQR)) age was 38 (34–43) years.

**Fig 1:**
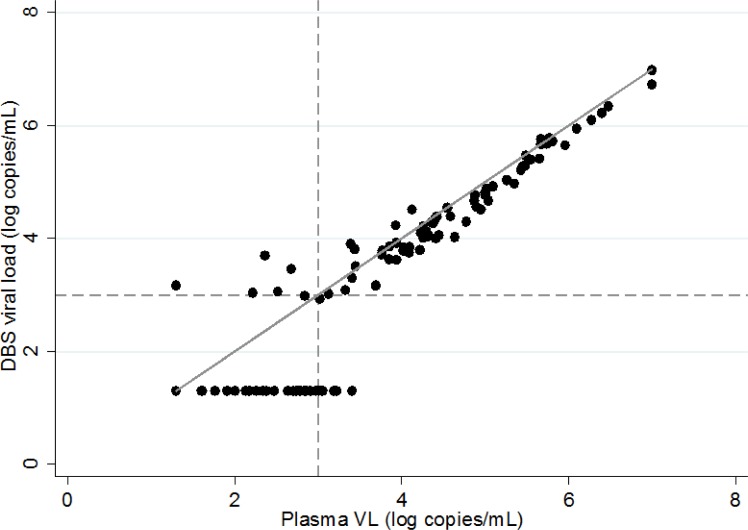
Correlation between plasma and DBS viral load measurements. The dashed line represents the threshold of 3 log copies/mL defining virological failure. The solid line represents the line of slope 1 displaying perfect concordance. Correlation: 0.95 (p<0.001).

**Fig 2:**
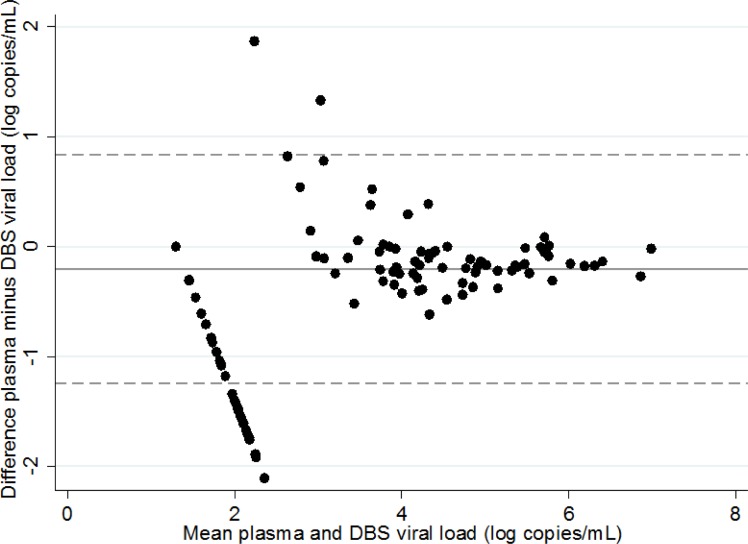
Bland-Altman analysis to evaluate concordance between plasma and DBS viral load measurements. The solid line is the mean difference between plasma and DBS viral load measurements and the dashed line is 95% confidence interval of the difference.

The plasma VL was below the detection limit in 104 (51.2%) patients. In patients with detectable plasma VL, the median (IQR) level was 4.01 (2.91–5.00) log copies/mL. DBS VL measurements were performed after a median (IQR) delay of conservation of 19 (12–26) days, and this delay never exceeded 27 days. DBS VL was below the detection limit in 132 (65.0%) patients. In patients with detectable DBS VL, the median (IQR) was 4.29 (3.79–5.21) log copies/mL. The correlation between plasma and DBS VL measurements was 0.95 (p<0.001) (Fig 1). The bland-Altman analysis showed overall good concordance between plasma and DBS VL measurements (Fig 2). The aligned dots at the bottom left of the graph were due to undetectable DBS VL measurements.

Out of the 203 patients, 71 (35.0%) had plasma VL above the threshold of 1000 copies/mL. Of these patients, 64 also had DBS VL above the threshold of 1000 copies/mL. This meant a sensitivity of 90.1% (95% CI: 80.7–95.9) (Tables 1 and 2). Downward classification was observed in 7 patients (plasma VL ≥1000 copies/mL but DBS VL <1000 copies/mL). In these 7 patients, the plasma VL ranged from 1023 copies/mL to 2570 copies/mL, and was <1200 copies/mL in 4 patients (S1 Table).

**Table 1:** Sensitivity and specificity of viral load measurements on DBS compared to plasma at the threshold of 1000 copies/mL.

	Plasma VL ≥1000 copies/mL	Plasma VL <1000 copies/mL	Total
DBS VL ≥1000 copies/mL	64 (90.1%)	5	69
DBS VL <1000 copies/mL	7	127 (96.2%)	134
Total	71	132	203

VL: viral load; DBS: dried blood spot

**Table 2:** Distribution of plasma and DBS VL measurements.

	Plasma VL Category (copies/mL), N (%)				
DBS VL Category (copies/mL), N (%)	<1000	1000–2999	3000–4999	≥5000	Total
<1000	127 (96.2)	7 (53.9)^a^	-	-	134
1000–2999	4 (3.0)^b^	3 (23.1)	1 (100)	-	8
3000–4999	1 (0.8)^b^	1 (7.7)	-	2 (3.5)	4
≥5000	-	2 (15.4)	-	55 (96.5)	57
Total	132	13	1	57	203

VL: viral load; DBS: dried blood spot

^a^ downward misclassification

^b^ upward misclassification

Of the 203 patients, 132 (65.0%) had plasma VL below the threshold of 1000 copies/mL. Of these patients, 127 also had a DBS VL below this threshold. This meant a specificity of 96.2% (95% CI: 91.4–98.8) (Tables 1 and 2). Upward misclassification was observed in 5 patients (S2 Table). In 4 of these patients, plasma VL was detected at a low level (range: 166 to 479 copies/mL) while it was above 1000 copies/mL in DBS (range: 1102 to 4934 copies/mL). Only one patient had an undetectable plasma VL and a DBS above 1000 copies/mL (1478 copies/mL).

At the threshold of 1000 copies/mL, concordance between plasma and VL results was 94.1% (95% CI: 89.9–96.9). The Kappa coefficient was 86.9% showing excellent concordance between the DBS and plasma VL measurements.

Increasing the DBS threshold to 3000 copies/mL, 60 of the 71 patients with plasma VL above 1000 copies/mL were in virological failure. This corresponded to a sensitivity of 84.5% (95% CI: 74.0–92.0) (Table 3). On the other hand, 131 out of 132 patients with plasma VL below 1000 copies/mL were considered in virological success. This corresponded to a specificity of 99.2% (95% CI: 95.9–100). Further increasing the DBS threshold to 5000 copies/mL decreased the sensitivity while the specificity reached 100% (Table 3). Lowering the DBS threshold to 839 copies/mL (limit of detection provided by manufacturer), sensitivity was 91.5% (95% CI: 82.5–96.8) and specificity was 95.5% (95% CI: 90.3–98.3) (Table 3).

**Table 3:** Sensitivity, specificity, misclassification, and Kappa coefficient by DBS testing and viral load thresholds on determining virological failure compared to plasma.

VL Threshold (copies/mL) Plasma: DBS	Sensitivity (%) (95% CI)	Specificity (%) (95% CI)	Upward misclassification (%) (95% CI)	Downward misclassification (%) (95% CI)	Kappa coefficient (95% CI)
1000: TND	95.8 (88.1–99.1)	63.6 (54.8–71.8)	36.3 (28.2–45.2)	4.2 (0.9–11.9)	51.8 (41.4–62.2)
1000 : 839^a^	91.5 (82.5–96.8)	95.5 (90.3–98.3)	4.5 (1.7–9.6)	8.5 (3.2–1.7)	87.0 (79.9–94.1)
1000: 1000	90.1 (80.7–95.9)	96.2 (91.4–98.8)	3.8 (1.2–8.6)	9.9 (4.1–19.3)	86.9 (79.7–94.1)
1000: 3000	84.5 (74.0–92.0)	99.2 (95.9–100)	0.8 (0–4.1)	15.5 (8.0–26.0)	86.6 (79.2–93.9)
1000: 5000	80.3 (69.1–88.8)	100 (97.2–100)	0 (0–2.8)	19.7 (11.2–30.8)	84.1 (76.2–92.0)

VL: viral load; DBS: dried blood spot; CI: confidence interval; TND: target not detected^a^ 839 copies/mL is the threshold provided by the manufacturer

### Impact of the duration on ART

To evaluate the impact of the ART duration on the DBS VL measurement, a stratified analysis was conducted considering patients on ART for <6 months on the one hand, and patients on ART for ≥6 months on the other hand. The ART duration could impact the quantity of blood cells harboring HIV and hence impact the specificity due to amplification of proviral DNA and/or intracellular RNA.

Restricting the analysis to the 39 patients on ART for <6 months, only 8 patients had a plasma VL ≥1000 copies/mL of whom 7 also had a DBS VL ≥1000 copies/mL. This corresponded to a sensitivity of 87.5% (95% CI: 47.3–99.7). The 31 other patients had a plasma VL <1000 copies/mL, of whom 27 had also a DBS VL <1000 copies mL. This corresponded to a specificity of 87.1% (95% CI: 70.1–96.4).

Considering the 124 patients on ART for ≥6 months, 27 had a plasma VL ≥1000 copies/mL of which 23 also had a DBS VL ≥1000 copies/mL. This corresponded to a sensitivity of 85.2% (95% CI: 66.3–95.6). The remaining 97 patients had a plasma VL <1000 copies/mL of whom 96 also had a DBS VL <1000 copies/mL. This corresponded to a specificity of 99.0% (95% CI: 94.3–100.0).

Specificity was significantly better in patients on ART for ≥6 months than in those on ART for <6 months (p = 0.012). Sensitivity however did not differ by ART duration (p = 0.99).

### Impact of the delay between DBS collection and VL measurements on DBS

DBS were kept at ambient temperature for two weeks and then frozen at -20°C until VL measurements were performed. To investigate the impact of storage conditions on DBS VL measurements, we performed an analysis stratified on non- frozen DBS on one hand, and on frozen DBS on the other hand, as freezing was thought to impact the sensitivity.

Considering the 77 patients for whom DBS were not frozen, 25 had a plasma VL ≥1000 copies/mL. Of these, 23 also had a DBS VL ≥1000 copies/mL. This meant a sensitivity of 92.0% (95% CI: 74.0–99.0). The remaining 52 patients had a plasma VL and a DBS VL <1000 copies/mL. This meant a specificity of 100% (95% CI: 93.2–100).

Considering the 126 patients for whom DBS were frozen, 46 had a plasma VL ≥1000 copies/mL. Of these, 41 had a DBS VL that was also ≥1000 copies/mL. This meant a sensitivity of 89.1% (95% CI: 76.4–96.4). The remaining 80 patients had a plasma VL <1000 copies/mL. Of these, 75 also had a DBS VL <1000 copies/mL. This meant a specificity of 93.8% (95% CI: 86.0–97.9).

Neither sensitivity (p = 0.56) nor specificity (p = 0.08) differed significantly based on the freezing status of the DBS.

## Discussion

This is the first field evaluation of the new CE marked optimized protocol developed by Abbott for VL quantification on DBS in routine clinical settings, in the laboratory responsible for VL monitoring in patients from the provinces of Northern Vietnam.

From a quantitative point of view, a very strong correlation was observed between plasma and DBS VL measurements. The estimated bias (mean difference between plasma and DBS VL measurements) was only 0.2 log copies/mL.

From a qualitative point of view, the capacity to detect virological failure at the threshold of 1000 copies/mL when using DBS was investigated through sensitivity and specificity which were 90.1% (95% CI: 80.7–95.9) and 96.2% (95% CI: 91.4–98.8), respectively. These results were comparable to those previously obtained when using venous whole blood collected on EDTA tubes, but using the previous two-spot Abbott DBS protocol [10, 11, 13, 15, 16].

With the new one-spot DBS protocol evaluated in the current study, sensitivity was 90.1% due to downward misclassification in 7 patients who presented DBS VL <1000 copies/mL. Of these 7 patients, DBS VL was detected in 4 (3 were below the detection limit of 839 copies/mL and 1 was 854 copies/mL) while their plasma VL was above the threshold of 1000 copies/mL, but the plasma VL level was modest as it ranged from 1043 to 1459 copies/mL. In the 3 remaining patients, DBS VL was not detected while their plasma VL was above the threshold of 1000 copies/mL (range: 1148 to 2570 copies/mL). Several explanations can be contemplated regarding these discrepancies. Firstly, inter-variability linked to the technique can’t be ruled out, especially for the samples which were detected on DBS, even if the Abbott technique has been described as having a low variability (<10% of variability) at low copy level [21]. Secondly, downward misclassification could also be explained by the deterioration of RNA in the DBS due to the freezing at -20°C after 15 days as recommended for HIV drug resistance genotyping [22]. Storage at -20°C, when using the Roche assay, has been suggested to deteriorate VL results [11]; the manufacturer now recommends not to freeze or refrigerate samples for extended period of time when using free virus elution (FVE) protocol. However, we did not find an impact of the storage at -20°C, a finding consistent with other studies [14]. Moreover, storage at -20°C was an alternative mentioned by the manufacturer (Abbott). It must be noted however that the DBS samples were not subject to repeated freeze-thaw cycles. Thirdly, the whole blood volume and the elution process could explain the discrepancies between plasma and DBS VL results [21, 23]. In the optimized DBS protocol, one 70 μL spot (instead of two 50 μL spots in the previous protocol) is diluted into 1.3 mL of lysis buffer. On the other hand, 0.8 mL of plasma is used for the plasma technique. It could also be linked to the use of Munktell TFN cards that possibly have a lower sensitivity for HIV RNA testing by comparison to the Whatman 903 filter papers [24].

Specificity of the DBS protocol was 96.2% and upward misclassification was observed in 5 patients who presented DBS VL above 1000 copies/mL while the plasma VL was below this threshold. In 4 of these patients, plasma VL were detected but at a low level (range: 166 to 479 copies/mL) while DBS VL ranged from 1102 to 4934 copies/mL. In the remaining patient, plasma VL was <40 copies/mL and the corresponding DBS VL was 1478 copies/mL. Importantly, patients with a VL under the threshold of 1000 copies/mL were classified as being in virological success whether or not VL is detected. Of these 5 upwardly misclassified patients, 4 would have received a therapeutic intervention (strengthening of ART adherence and ART modification or intensification) following European or American guidelines due to the viral replication, even if it is at a low level to prevent therapeutic failure and HIV drug resistance acquisition [25–27]. Thus, the impact of the DBS results would have been beneficial for 4 of these 5 patients.

Upward misclassification could be due to amplification of proviral DNA and intracellular RNA contaminations [28, 29] especially when HIV DNA reservoir is high [30]. It must be noted that out of the 5 patients with upward misclassification, 4 were on ART for less than 6 months while the remaining one was on ART for more than 6 months. Stratifying the analysis by ART duration showed a significantly better specificity on those on ART for more than 6 months. Although the patients on ART for less than 6 months presented low plasma VL, they may still present a higher level of cells harboring HIV. In these patients, discrepancies could be explained by the amplification of intracellular RNA.

To perform this DBS evaluation, patients not yet on ART, on ART for less than 6 months, and on ART for more than 6 months were enrolled. These three groups of patients allowed us to obtain a large range of VL values from undetectable to very high. Other studies which have evaluated DBS VL performance also enrolled HIV negative patients [11]. This choice would de facto have cancelled the potential impact of proviral DNA and intracellular RNA amplification and could have artificially improve specificity performance. The choice of patients enrolled also allowed the study to be close to the real–life situation in terms of a continuum of VL levels. Had this evaluation been performed on patients with undetectable VL on one hand, and on high VL patients on the other hand, DBS performances would have been even better. In view of the extremely high quantitative correlation and given the VL levels when discrepancies were observed, one could imagine that 100% sensitivity and specificity could have been achieved.

From a clinical perspective, a downward misclassification (default in sensitivity) means that some patients who are in virological failure are not identified when using DBS. This is an important issue as following the WHO guidelines would mean that these patients would not be virologicaly evaluated for another 12 months or until an immunological or clinical deterioration is observed, and would be maintained on an inefficient ART regimen, thus increasing the likelihood of HIV drug resistance acquisition. An upward misclassification means that patients are incorrectly categorized as being in virological failure and that unnecessary switches to second line ART could occur. However, following current WHO guidelines [5], patients with a VL ≥1000 copies/mL undergo counselling to improve ART adherence and a VL measurement is performed one to three month later. Thus, the probability of having two consecutive DBS VL above 1000 copies/mL, measured inaccurately, is low. Moreover, only patients on ART for more than 6 months would be evaluated, limiting the impact of proviral DNA and intracellular RNA amplification. A small default in specificity is less worrisome, other than the additional costs of unnecessary VL testing in a small proportion of patients. However, the high specificity obtained in our study and in others [10, 13, 16] limits this risk. When both sensitivity and specificity reach acceptable levels, this motivates maximization of the sensitivity (minimization of downward misclassification).

One strength of this study lies in its setting in field conditions, in a routine laboratory in charge of the VL monitoring of HIV infected patients on ART from Northern provinces of Vietnam, and not in a research-dedicated laboratory. Duration and conditions of DBS storage also reflect field conditions. It was important to perform this evaluation in these conditions, in order to make sure that scaling-up of VL monitoring on DBS is possible. Strictly limiting this evaluation on HIV positive patients also strengthens our results and allows good extrapolation. Finally, all DBS VL results were obtained blinded from plasma VL results.

One limitation of our study is the DBS preparation at the laboratory in charge of VL monitoring and not at the outpatient clinic. This allowed maximizing the quality of the plasma sample, used as the gold standard. However, venous sampling and then spotting of whole blood with a calibrated pipette is easy to implement in laboratories in remote areas. Another limitation was the use of convenience sampling which does not allow for estimating the prevalence of failure, and does not allow for estimating the positive and negative predictive values. For the quantitative evaluation, we chose to keep all measurements [10, 14, 15] and arbitrarily attributed the value of 20 copies/mL for all undetectable VL measurements, whether on plasma or on DBS. This allowed matching observations which were both undetectable, although this also introduced over-estimated differences when plasma VL was detectable. Despite this, correlation between plasma and DBS VL measurements was very high and the bias was reduced. Attributing a similar value to all VL measurements <839 copies/mL, in plasma and from DBS, would have smoothed differences and somewhat artificially improved the correlation. It must also be noted that the goal of this study was to evaluate the ability of DBS to diagnose virological failure at the threshold of 1000 copies/mL, and not to perform a quantitative evaluation.

In conclusion, in settings where plasma VL monitoring is not feasible, the new CE marked Abbott optimized protocol, offers the opportunity to efficiently monitor HIV patients on ART with good performances at the current threshold of 1000 copies/mL. Scaling up of VL monitoring using DBS can contribute to efforts to reach the last 90 the UNAIDS targets of 90-90-90 to help end the AIDS epidemic.

## Supporting information

S1 Table Downward misclassification. Seven patients with plasma VL >1000 copies/mL but DBS VL <1000 copies/mL.
(DOCX)

S2 Table Upward misclassification. Five patients with plasma VL <1000 copies/mL but DBS VL >1000 copies/mL.
(DOCX)
