# Supplementary material for: First field evaluation of the optimized CE marked Abbott protocol for HIV RNA testing on dried blood spot in a routine clinical setting in Vietnam
Source: PLoS One. 2018 Feb 9;13(2):e0191920. doi: 10.1371/journal.pone.0191920 (PMC5806875; doi:10.1371/journal.pone.0191920)
Supplement: S1 Table — Seven patients with plasma VL >1000 copies/mL but DBS VL <1000 copies/mL. (DOCX) [file pone.0191920.s001.docx]

| **Plasma VL (copies/mL)** | **DBS VL (copies/mL)** |
| --- | --- |
| 1148 | Target not detected |
| 1047 | 854 |
| 1660 | Target not detected |
| 2570 | Target not detected |
| 1549 | <839 |
| 1096 | <839 |
| 1023 | <839 |

VL: viral load; DBS; dried blood spots
