# Supplementary material for: First field evaluation of the optimized CE marked Abbott protocol for HIV RNA testing on dried blood spot in a routine clinical setting in Vietnam
Source: PLoS One. 2018 Feb 9;13(2):e0191920. doi: 10.1371/journal.pone.0191920 (PMC5806875; doi:10.1371/journal.pone.0191920)
Supplement: S2 Table — Five patients with plasma VL <1000 copies/mL but DBS VL >1000 copies/mL. (DOCX) [file pone.0191920.s002.docx]

| **Plasma VL (copies/mL)** | **DBS VL (copies/mL)** |
| --- | --- |
| 229 | 4934 |
| 166 | 1102 |
| 331 | 1146 |
| 479 | 2873 |
| <40 copies/mL | 1478 |

*4 of these patients were on ART for <6 months. These patients may have a larger number of cells harboring HIV leading to amplification of viral DNA thus explaining the higher level in DBS*

VL: viral load; DBS; dried blood spots
